# Editorial: Biomarkers of neonatal brain injury

**DOI:** 10.3389/fped.2023.1271564

**Published:** 2023-08-29

**Authors:** Sven Wellmann, Deirdre M. Murray, Kasper Jacobsen Kyng

**Affiliations:** ^1^ Department of Neonatology, University Children’s Hospital Regensburg (KUNO), Hospital St. Hedwig of the Order of St. John, University of Regensburg, Regensburg, Germany; ^2^ Department of Paediatric and Child Health, University College Cork, Cork, Ireland; ^3^ Department of Paediatrics, Institute of Clinical Medicine, Aarhus University Hospital, Aarhus, Denmark

**Keywords:** biomarker, neonatal brain injury, hypoxic-ischemic encephalopathy (HIE), diagnostic tools, prognostic indicators, neonatal encephalopathy (NE)

**Editorial on the Research Topic** Biomarkers of neonatal brain injury

**Figure 1:**
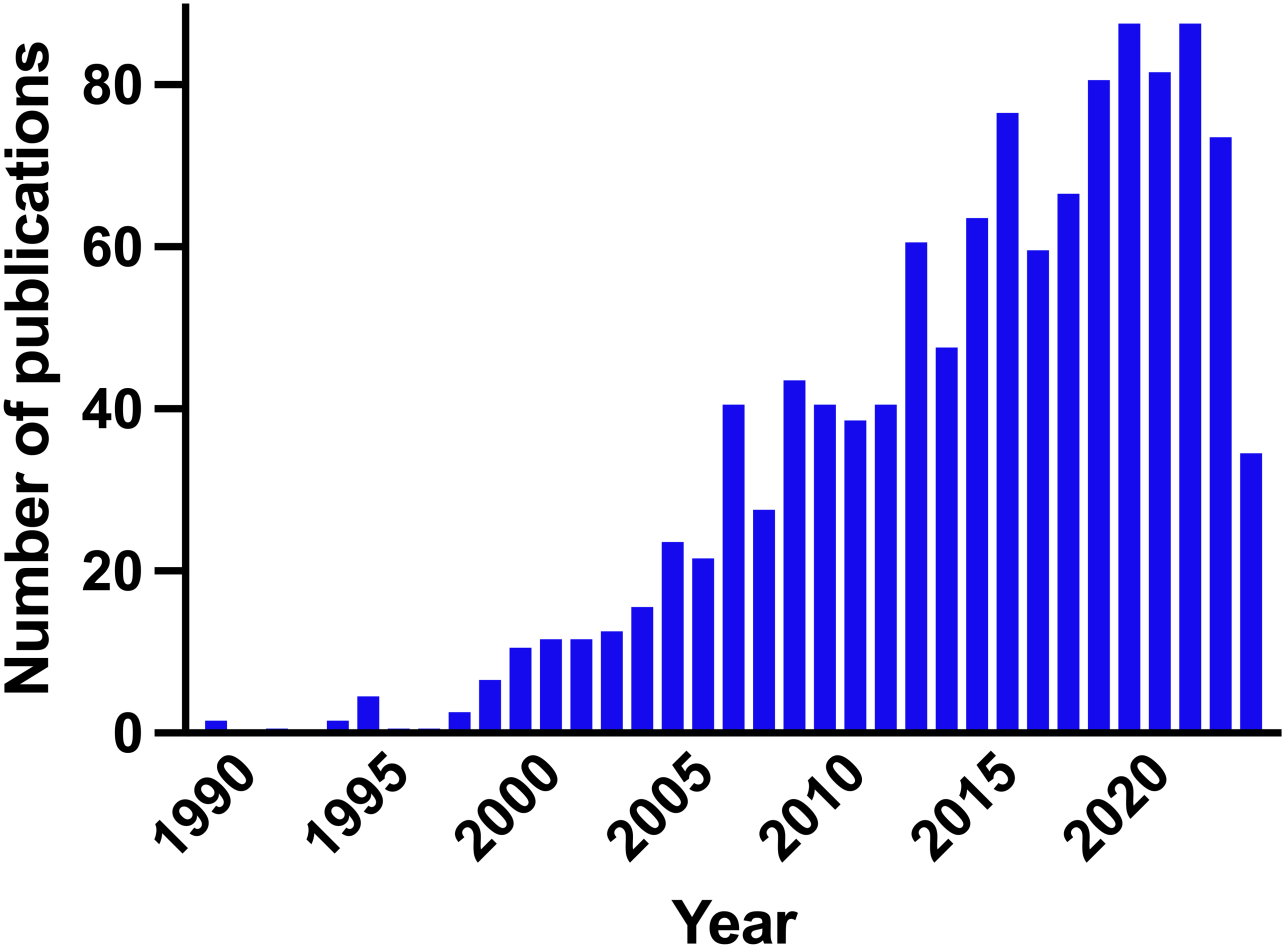
Number of publications per year on biomarkers of neonatal brain injury in newborn infants. PubMed search query: ((Biomarker) AND (Brain injury) AND (Newborn OR Preterm OR Infant)).

Diagnosing brain injury in newborns is crucial because early intervention can significantly impact long-term development and overall quality of life. The brain undergoes rapid development during the neonatal period, and any injury to the brain can disrupt this delicate process. Neurodevelopment is a major concern in several conditions including preterm birth, intrauterine growth restriction, periventricular leukomalacia, white matter injury, neonatal encephalopathy (NE), hypoxic-ischemic encephalopathy (HIE), and neonatal stroke. Premature infants are particularly susceptible to brain injury due to their underdeveloped organs and fragile systems. Although survival rates of infants born extremely preterm continue to increase (1), one-third of children born extremely preterm still suffer moderate to severe neurodevelopmental disabilities at school age (2). At term, NE affects 0.5–3/1,000 live births in high-income countries, and more in low- and middle-income countries, affecting millions of babies worldwide (3). Diagnosing brain injury in newborns can be challenging because the symptoms may not be immediately apparent or may be non-specific. Advanced imaging techniques, such as cranial ultrasound, magnetic resonance imaging (MRI), and electroencephalography (EEG), are often utilized to aid in the diagnosis. However, not all NICUs have access to these resources, which can further complicate early identification. This highlights the need for effective diagnostic and treatment strategies in NICUs. Prompt diagnosis allows healthcare professionals to identify the extent and location of the injury and opens up treatment possibilities enabling timely intervention to minimize potential long-term consequences. Biomarkers of brain injury hold the potential to significantly improve the management of newborns at risk of brain injury. The number of publications on biomarkers of neonatal brain injury has been steadily increasing since the early 2000s (Figure 1). So far though, no proposed biomarkers have been validated to be better than clinical assessment of NE for identifying infants who will likely benefit from neuroprotective treatments (4). The goals and challenges are overlapping for different classes of biomarkers. Unsolved issues include reference values in different body fluids, the use of single vs. serial assessment, and panels of markers. Recent studies have explored the potential of biomarkers in predicting and understanding neonatal brain injury, providing valuable insights into diagnostic and prognostic tools. This editorial aims to summarize four notable papers that contribute to the evolving field of neonatal brain injury biomarkers.

Pavel et al. conducted a cohort study to assess heart rate variability (HRV) as a potential biomarker for predicting the severity of EEG grade in neonates with HIE within the first 12 h of birth. The study included 120 infants with HIE, and HRV features were analyzed alongside clinical parameters. The results demonstrated that early HRV, combined with clinical information, accurately predicted EEG grade, providing valuable objective information on HIE severity when EEG monitoring was not available in the early postnatal period. HRV was analyzed using custom software and manual artifact detection in a process that would need to be simplified or automated prior to bedside use.

Kyng et al. investigated the potential of neurofilament light chain (NfL) as a biomarker for brain injury in a newborn piglet model of hypoxia-ischemia. The study analyzed serum NfL levels in 48 piglets subjected to hypoxic-ischemic insults, along with brain imaging and histologic assessments. The results revealed a significant increase in NfL levels after hypoxia-ischemia, with the highest values observed at 72 h post-insult. NfL levels at 72 h were predictive of a poor outcome on cortical histopathology and magnetic resonance spectroscopy basal ganglia Lac/NAA ratio, indicating its potential as a sensitive and specific biomarker for moderate–severe HIE.

Balog et al. conducted a prospective, observational study to analyze cardiac function parameters using non-invasive hemodynamic monitoring in neonates with moderate–severe HIE undergoing therapeutic hypothermia (TH). The study included 26 neonates, and heart rate (HR), stroke volume (SV), and cardiac output (CO) data were collected throughout TH and rewarming. The findings demonstrated that patients with adverse outcomes had lower SV and higher HR during TH, indicating differences in cardiac function associated with neurodevelopmental outcomes. HR during hypothermia was found to be independently associated with neurodevelopmental outcomes, emphasizing its relevance in guiding cardiovascular supportive therapy.

Efstathiou et al. explored the kinetics of circulating progenitor cells (CPCs) in preterm neonates with encephalopathy, aiming to understand the brain's endogenous regeneration processes after injury. The study enrolled 47 preterm neonates and analyzed peripheral blood samples using flow cytometry to assess CPCs, along with brain injury biomarkers and clinical factors. The results indicated an enhanced mobilization of CPCs following brain injury, suggesting the presence of an endogenous brain regeneration process. The findings contribute to understanding the related pathophysiology and highlight the potential of CPCs as biomarkers for evaluating brain injury and predicting outcomes.

Exploring the utility of biomarkers across different neonatal brain injuries can provide valuable insights into shared mechanisms and pathways. Investigating biomarkers that have the potential for cross-disease validation may lead to the identification of common therapeutic targets and facilitate the development of targeted interventions for multiple conditions. The studies discussed above highlight the progress made in this field, focusing on diverse biomarkers including physiological, biochemical, hemodynamic, and functional biomarkers. Incorporating these biomarkers into clinical practice has the potential to enhance early detection, risk stratification, and individualized management of neonates at risk of brain injury. Challenges for the future include the development and validation of biomarkers that can predict acute injury, cross-disease validation of biomarkers, validation of previous findings in larger cohorts, and establishing correlations with long-term neurodevelopment to assist in personalized care. Most likely not a single biomarker will make the diagnostic breakthrough but the intelligent combination of many. Thus, further research and validation are warranted to establish the utility of these biomarkers and their integration into routine neonatal care, ultimately leading to improved neurodevelopmental outcomes for affected infants.
